# Effect of Metabolic Syndrome and Individual Components on Colon Cancer Characteristics and Prognosis

**DOI:** 10.3389/fonc.2021.631257

**Published:** 2021-03-04

**Authors:** Ana Silva, Sofia S. Pereira, Mariana P. Monteiro, António Araújo, Gil Faria

**Affiliations:** ^1^ Pharmacy Department, Centro Hospitalar Universitário do Porto, Porto, Portugal; ^2^ School of Health, Polytechnic Institute of Porto, Polytechnic of Porto, Porto, Portugal; ^3^ Endocrine, Cardiovascular & Metabolic Research, Unit for Multidisciplinary Biomedical Research (UMIB) of Institute of Biomedical Sciences Abel Salazar (ICBAS), University of Porto, Porto, Portugal; ^4^ Centre for Obesity Research, University College London, London, United Kingdom; ^5^ Unit of Oncobiology Research, Unit for Multidisciplinary Biomedical Research (UMIB) of Institute of Biomedical Sciences Abel Salazar (ICBAS), University of Porto, Porto, Portugal; ^6^ Medical Oncology Department, Centro Hospitalar Universitário do Porto, Porto, Portugal; ^7^ iGo Department, CINTESIS-Center for Research in Health Technologies and Information Systems, Porto, Portugal; ^8^ General Surgery, Hospital de Pedro Hispano – Unidade Local de Saúde de Matosinhos, Senhora da Hora, Portugal; ^9^ Department of Surgery, Faculty of Medicine, University of Porto, Porto, Portugal

**Keywords:** colon cancer, metabolic syndrome, tumor staging, survival, lymph node ratio

## Abstract

Metabolic syndrome (MS) is recognized as a risk factor for colon cancer (CC). However, whether the cluster of metabolic changes that define MS also influence CC prognosis remains unclear. Thus, our aim was to investigate whether the presence of MS or any of the MS individual components could provide prognostic information on tumor phenotype and survival outcomes. Clinical and pathological data from patients with CC (n = 300) who underwent surgical resection at a single tertiary hospital were retrospectively collected to evaluate presence of MS components and diagnostic criteria, CC phenotype and disease outcomes. Patients were allocated into two groups according to the presence or absence of MS (n = 85 MS vs n = 83 non-MS). The overall prevalence of MS individual components was 82.7% for increased waist-circumference (WC), 61.3% for high blood pressure (BP), 48.8% for low HDL-cholesterol, 39.9% for high fasting glucose, and 33.9% for hypertriglyceridemia. Patients in the MS group presented smaller tumors (p = 0.006) with lower T-stage (p = 0.002). High BP (p = 0.029) and hypertriglyceridemia (p = 0.044) were associated with a smaller tumor size, while low-HDL (p = 0.008) was associated with lower T-stage. After propensity score matching using age, tumor size and staging as covariates high-BP (p = 0.020) and WC (p = 0.003) were found to influence disease-free survival, but not overall survival. In conclusion, despite MS being an established risk factor for CC, our data does not support the hypothesis that MS components have a negative impact on disease extension or prognosis. Nevertheless, a protective role of BP and lipid lowering drugs cannot be excluded.

## Introduction

Colorectal cancer (CRC) is the third most common malignancy worldwide with approximately 2 million new cases diagnosed in 2018 (1). Therefore, identifying clinically relevant prognostic factors that could be used as a guidance for tailoring therapeutic decisions should be considered a priority.

Abdominal obesity was previously identified as one of the main risk factors for colon cancer (CC) (2). Indeed, excess visceral adiposity is often associated with a chronic low grade inflammatory state characterized by increased production of inflammatory cytokines and adipocytokines, which are responsible for inducing several metabolic dysfunctions with carcinogenic potential (2). Thus, the interest in characterizing the extent of the impact of metabolic changes on cancer development and progression has gradually increased; and most particularly focusing on metabolic syndrome (MS) as a risk factor for CC (3, 4).

MS is a cluster of risk factors that includes abdominal obesity, high blood-pressure (BP), dysglycemia or type 2 diabetes mellitus, hypertriglyceridemia, and low high-density lipoprotein cholesterol (HDL-c) (5), all of which have also been independently associated with an increased risk of cancer (2, 4, 6). Despite substantial interest in the relationship between MS and CC, whether this cluster of metabolic abnormalities also influences tumor characteristics and mortality remains unclear. Thus, we aimed to further investigate whether MS or any of the MS individual components could provide prognostic information for tumor characteristics and survival outcomes. Since MS risk factors can be easily assessed in routine clinical practice, if a prognostic value could be identified, this would be clinically valuable to guide oncologists on therapeutic and lifestyle interventions that might modify disease progression.

## Materials and Methods

### Patients and Study Protocol

Data on all patients diagnosed with colon adenocarcinoma that underwent surgical resection at a single tertiary public institution between January 2010 and December 2015 were retrospectively reviewed. Detailed clinical information retrieved from electronic medical records, included clinical presentation, co-morbidities, laboratory findings required for MS diagnosis, such as lipid profile and fasting blood glucose, and pathological findings required for CC tumor staging.

### Metabolic Syndrome Criteria

Patients were classified as having MS whenever at least 3 out of the 5 individual components of the Harmonized Criteria were present, namely: (i) abdominal obesity (waist circumference (WC) ≥94 cm (male) or ≥80 cm (female) (Europid)); (ii) elevated triglycerides (>150 mg/dL) or ongoing treatment with triglyceride lowering drugs; (iii) low HDL-c (<40 mg/dL (males) and <50 mg/dL (females) or ongoing treatment with HDL-c raising drugs; (iv) high BP (systolic ≥130 and/or diastolic ≥85 mm Hg) or ongoing treatment with antihypertensive drugs; (v) fasting blood glucose ≥100 mg/dL or ongoing treatment with glucose lowering drugs (5).

### Colon Cancer Tumor Staging

Data on tumor pathological characteristics including primary tumor location, larger tumor diameter, pathological stage, and presence of lymph, vascular and peri-neural invasion were retrieved from the electronic clinical records system. Primary tumor location was considered as right-sided for tumors involving from the cecum to the transverse colon and as left-sided for tumors involving from the splenic flexure to sigmoid. Colon cancer staging was performed according to the American Joint Committee on Cancer (AJCC) criteria and the Duke’s staging system.

Lymph node ratio (LNR) was calculated as the ratio between the number of metastatic and dissected lymph nodes (LN). Patients with node-negative disease were classified as LNR=0%. Patients with node-positive disease were classified into LNR categories defined according to the 50th percentile of patients with node-positive disease, as previously described (7).

Patient follow-up data was retrieved until death or last visit. Overall survival (OS) was defined as the time interval from the date of diagnosis to the date of death. Recurrence-free survival (RFS) was defined as the interval from the date of diagnosis to the date of tumor recurrence or date of last follow-up. Disease-free survival (DFS) was defined as the time interval from the date of diagnosis to date of tumor recurrence, death or date of last follow-up.

This study protocol including clinical data accession was granted approval by the Institutional Ethics Review Board (2015.178(153-DEFI/142-CES).

### Statistical Analysis

Continuous variables were expressed as mean ( ± standard deviation, SD) or median (interquartile range (IQR)), according to data distribution. Normality of the variables was determined by the Shapiro-Wilk test. Categorical variables were expressed as counts and proportions and compared with Chi-square test. Student’s t-test and Mann-Whitney U-test were used to evaluate differences between groups in continuous variables, according to data distribution.

Propensity score matching between patients in the MS and no-MS group was conducted to obtain matched data to reduce the influence of data deviation and confounding variables in survival analysis. Propensity scores were estimated accounting for the following patient parameters: age, tumor size and TNM staging. Matching was conducted by a 1:1 Mahalanobis distance optimal matching within caliper set to 0.25 standard deviations of the logit of the propensity score, as suggested by Rosenbaum and Rubin (8). The quality of matching was evaluated by computing the standardized difference in means for the two groups before and after matching. Statistical analysis for recurrence and survival rates was determined by the Kaplan–Meier method. The log-rank test was used for the comparison of survival between patients’ groups. Cox proportional hazard models were used to explore associations of MS and individual components with survival outcomes. Multivariate analysis using the Cox model was performed for all variables found to be significant in the univariate analysis. Statistical significance was considered for p<0.05. Data was stored and analyzed using SPSS Statistics 25.0 (SPSS Inc., Chicago, IL, USA) and XLSTAT 2020.5.1 (Addinsoft Inc., New York, USA).

## Results

Detailed clinical data was retrieved from patients (n=300) that underwent colon cancer surgical resection at a single public hospital institution. Participants were excluded whenever the histological diagnosis was other than colon adenocarcinoma (n=36), the histological diagnosis was carcinoma *in situ* (n=5), the information was incomplete to allow tumor staging (n=30), information on any of the MS components was unavailable (n=37), patients have had any anti-neoplastic treatment prior to first evaluation at the hospital institution (n=17) or were lost to follow-up (n=7). After exclusions, one hundred and sixty-eight patients (n=168) were included in the analysis. The subjects were divided into two groups based on the presence or absence of MS (n= 85 with MS vs n= 83 without MS). The baseline characteristics of the patients are shown in **Table 1**. Patients with MS were predominantly male (51.8%) and significantly older (74 vs. 67 years old; p<0.001). MS patients also had a significantly larger waist circumference when compared with patients without MS (105.6 vs. 101.2 cm; p<0.001). The overall prevalence of MS components was 82.7% for elevated WC, 61.3% for high BP, 48.8% for low HDL-c, 39.9% for dysglycemia, and 33.9% for hypertriglyceridemia. No significant differences in red blood cell (RBC) counts, hematocrit or hemoglobin between study groups were observed (**Table 1**).

**Table 1:** Patient’s characteristics according to metabolic syndrome status.

	All patients (n=168)	No MS (n=83)	MS (n=85)	p value
Age (years), mean (SD)	70 (12)	67 (14)	74 (9)	<0.001
Male sex, n (%)	98 (58.3)	54 (65.1)	44 (51.8)	0.081
WC (cm), median (IQR)	102.4 (95.0–110.4)	101.2 (90.0–106.7)	105.6 (99.3–115.4)	<0.001
MS risk factors, median (IQR)	3 (2–4)	2 (1–2)	4 (3–5)	<0.001
Dysglycemia, n (%)	67 (39.9)	11 (13.3)	56 (65.9)	<0.001
Low HDL-c, n (%)	82 (48.8)	10 (12.0)	72 (84.7)	<0.001
High BP, n (%)	103 (61.3)	29 (34.9)	74 (87.1)	<0.001
Hypertriglyceridemia, n (%)	57 (33.9)	4 (4.8)	53 (62.4)	<0.001
Elevated WC, n (%)	139 (82.7)	60 (72.3)	79 (92.9)	<0.001
RBC (x10^12/L), median (IQR)	4.5 (3.9–4.8)	4.6 (4.1–4.9)	4.4 (3.9–4.7)	0.059
Hematocrit (%), mean (SD)	37.2 (0.5)	37.6 (0.7)	36.8 (0.6)	0.424
Hemoglobin (g/dL), mean (SD)	12.1 (0.2)	12.2 (0.3)	11.9 (0.2)	0.355
Tumor location, n (%)				
Left side	100 (59.5)	53 (63.9)	47 (55.3)	0.258
Right side	68 (40.5)	30 (36.1)	38 (44.7)	
Tumor size (cm), median (IQR)	4.0 (3.0–5.5)	4.5 (3.5–5.5)	4.0 (3.0–5.0)	0.006
T stage, n (%)				
1/2	42 (25.0)	12 (14.5)	30 (35.3)	0.002
3/4	126 (75.0)	71 (85.5)	55 (64.7)	
N stage, n (%)				
0	102 (60.7)	48 (57.8)	54 (63.5)	0.185
1	42 (25.0)	19 (22.9)	23 (27.1)	
2	24 (14.3)	16 (19.3)	8 (9.4)	
Metastatic LN, median (IQR)	0 (0–2)	0 (0–3)	0 (0–1)	0.277
Retrieved LN, median (IQR)	14 (10–21)	16 (11–23)	14 (10–18)	0.052
LNR, median (IQR)	0.15 (0.08–0.36)	0.22 (0.09–0.36)	0.12 (0.07–0.36)	0.159
LNR, n (%)				
LNR = 0%	103 (61.3)	49 (59.0)	54 (63.5)	0.332
LNR <15%	32 (19.0)	14 (16.9)	18 (21.2)	
LNR ≥15%	33 (19.6)	20 (24.1)	13 (15.3)	
M stage, n (%)				
0	135 (80.4)	66 (79.5)	69 (81.2)	0.787
1	33 (19.6)	17 (20.5)	16 (18.8)	
AJCC Classification				0.285
Dukes Classification, n (%)				
A	36 (21.4)	11 (13.3)	25 (29.4)	0.070
B	48 (28.6)	28 (33.7)	20 (23.5)	
C	52 (31.0)	28 (33.7)	24 (28.2)	
D	32 (19.0)	16 (19.3)	16 (18.8)	
Lymphatic Invasion, n (%)	86 (51.2)	44 (53.0)	42 (49.4)	0.641
Vascular Invasion, n (%)	65 (38.7)	38 (45.8)	27 (31.8)	0.062
Perineural Invasion, n (%)	49 (29.2)	28 (33.7)	21 (24.7)	0.198
Relapse, n (%)	46 (27.4)	24 (52.2)	22 (47.8)	0.659
Death, n (%)	45 (26.8)	21 (46.7)	24 (53.3)	0.668

Continuous variables are presented as mean ( ± SD) or median (IQR). Categorical data are presented as n (%). BP, Blood Pressure; IQR, Interquartile range; LN, lymph node; LNR, lymph node ratio; MS, metabolic syndrome; RBC, Red Blood Cells; SD, Standard deviation; WC, waist circumference.

No associations were found between MS and most of the clinical or pathological variables assessed in this study, including primary tumor location (p=0.258), lymph (p = 0.641), vessel (p = 0.062), or neural (p=0.198) invasion nor distant metastatic disease (p = 0.787) (**Table 1**). No residual tumor was found after primary tumor resection in all patients (R0). Patients in the MS group had less advanced T-stage (p = 0.002) and a significantly smaller median tumor size than the patients in the no-MS group (4.0 cm vs. 4.5 cm; p = 0.006) (**Table 1**). No differences were found between groups regarding the disease stage by the AJCC (p = 0.285) (**Supplementary Table 1**) nor Dukes (p = 0.070) classification (**Table 1**). The median LN harvested during surgery was 14 (range, 3–74), with no significant differences between the groups despite the marginally lower LN number in the MS group (14 vs. 16; p = 0.052). Median LNR of node-positive patients was 0.15, setting LNR threshold at 15%. LNR categories were stablished according to the extension of LN involvement in LNR<15% and LNR≥15%. No association was found between MS and LNR (p = 0.332) or metastatic LN (p = 0.277) (**Table 1**).

The effect of each MS component in tumor characteristics revealed that high BP (p=0.029) and hypertriglyceridemia (p=0.044) were associated with a smaller tumor size (**Table 2**), while low-HDL (p=0.008) was associated with lower T-stage (**Table 3**).

**Table 2:** Association of individual components of MS with tumor size.

MS Component	Tumor size (cm)	p value
Dysglycemia, median (IQR)		0.946
Yes	4.2 (3.0–5.5)	
No	4.0 (3.1–5.3)	
Low HDL-c, median (IQR)		0.229
Yes	4.0 (3.0–5.5)	
No	4.5 (3.5–5.5)	
High BP, median (IQR)		**0.029**
Yes	4.0 (3.0–5.0)	
No	4.5 (3.5–5.5)	
Hypertriglyceridemia, median (IQR)		**0.044**
Yes	4.0 (3.0–5.0)	
No	4.5 (3.5–5.5)	
Elevated WC, median (IQR)		0.365
Yes	4.0 (3.0–5.5)	
No	4.1 (3.5–5.8)	

BP, Blood pressure; IQR, Interquartile range; MS, Metabolic Syndrome; WC, Waist circumference. In bold: p-values with statistical significance.

**Table 3:** Association of individual components of MS with T-stage.

MS Component	T-stage		p value
	1/2	3/4	
Dysglycemia, n (%)			0.056
Yes	20 (47.6)	81 (64.3)	
No	22 (52.4)	45 (35.7)	
Low HDL-c, n (%)			**0.008**
Yes	14 (33.3)	72 (57.1)	
No	28 (66.7)	54 (42.9)	
High BP, n (%)			0.410
Yes	14 (33.3)	51 (40.5)	
No	28 (66.7)	75 (59.5)	
Hypertriglyceridemia, n (%)			0.074
Yes	23 (54.8)	88 (69.8)	
No	19 (45.2)	38 (30.2)	
Elevated WC, n (%)			0.125
Yes	4 (9.5)	25 (19.8)	
No	38 (90.5)	101 (80.2)	

BP, Blood pressure; MS, Metabolic Syndrome; WC, Waist circumference. In bold: p-values with statistical significance.

**Figure 1:**
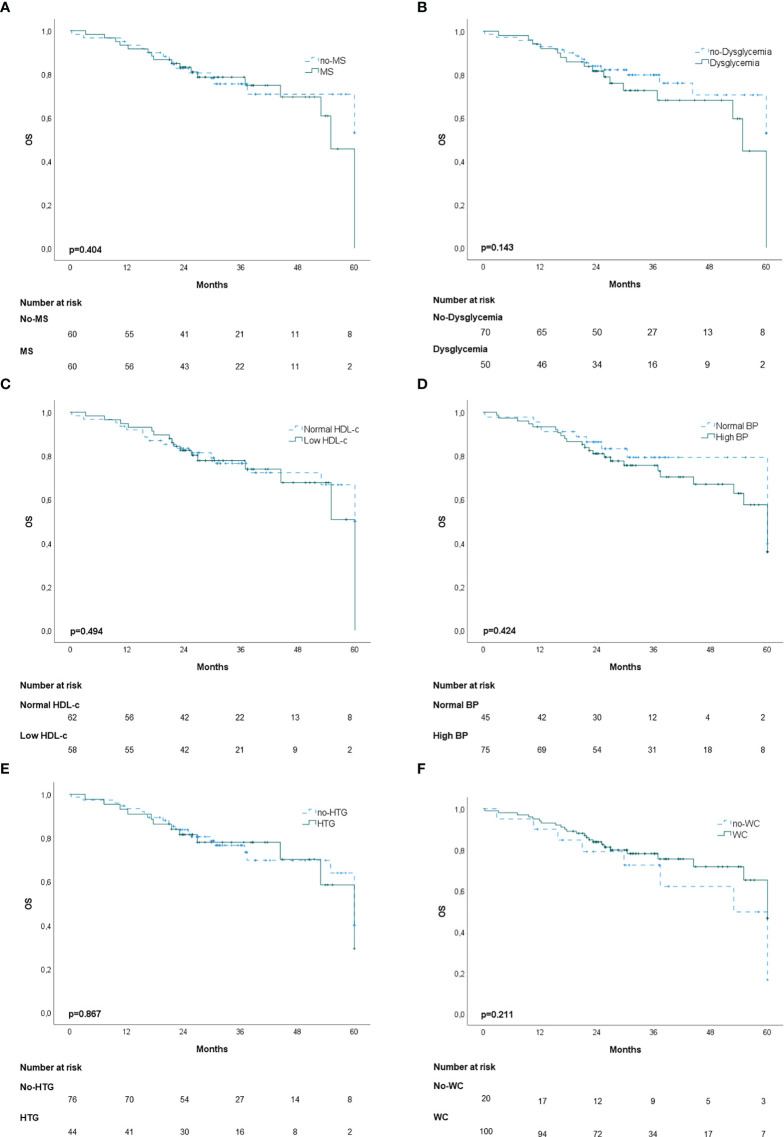
Kaplan-Meier overall survival (OS) curves: **(A)** patients with and without metabolic syndrome (MS); **(B)** patients with and without dysglycemia (fasting plasma glucose ≥100 mg/dl); **(C)** patients with and without low HDL-c; **(D)** patients with and without high blood pressure (BP); **(E)** Patients with and without hypertriglyceridemia; **(F)** patients with and without elevated waist-circumference (WC).

**Figure 2:**
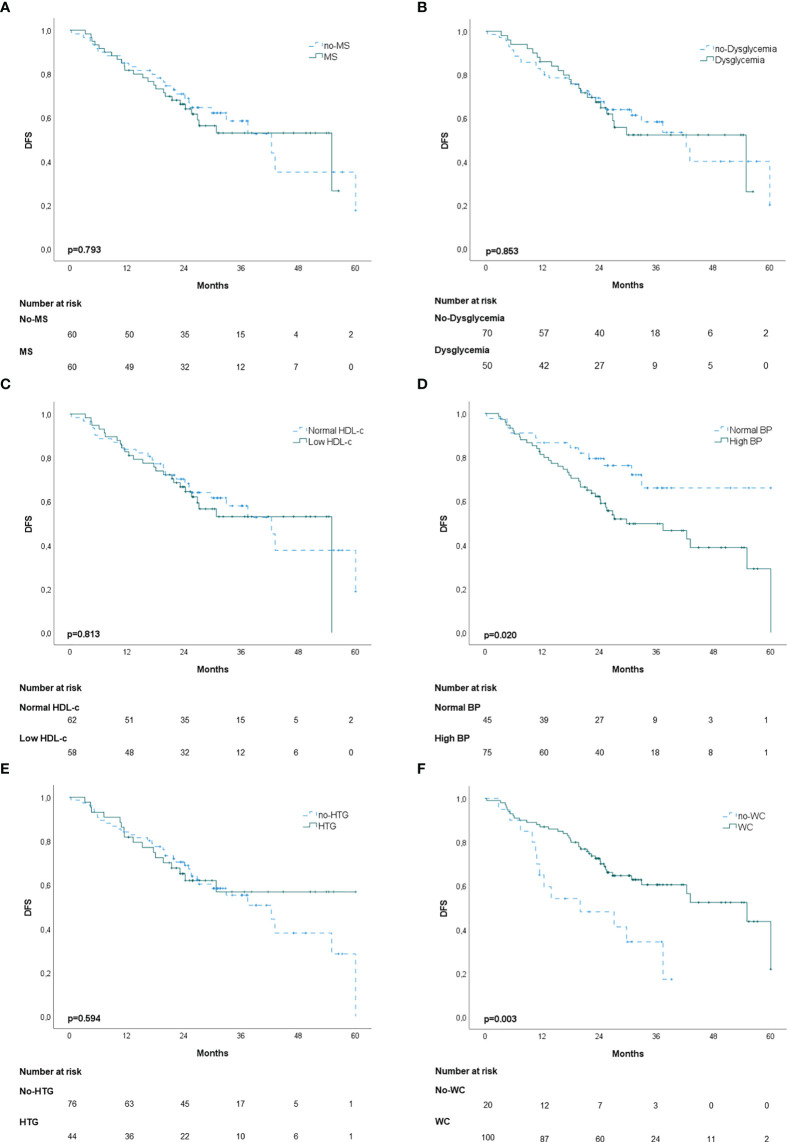
Kaplan-Meier disease-free survival curves (DFS): **(A)** patients with and without metabolic syndrome (MS); **(B)** patients with and without dysglycemia (fasting plasma glucose ≥100 mg/dl); **(C)** patients with and without low HDL-c; **(D)** patients with and without high blood pressure (BP); **(E)** patients with and without hypertriglyceridemia; **(F)** patients with and without elevated waist-circumference (WC).

**Figure 3:**
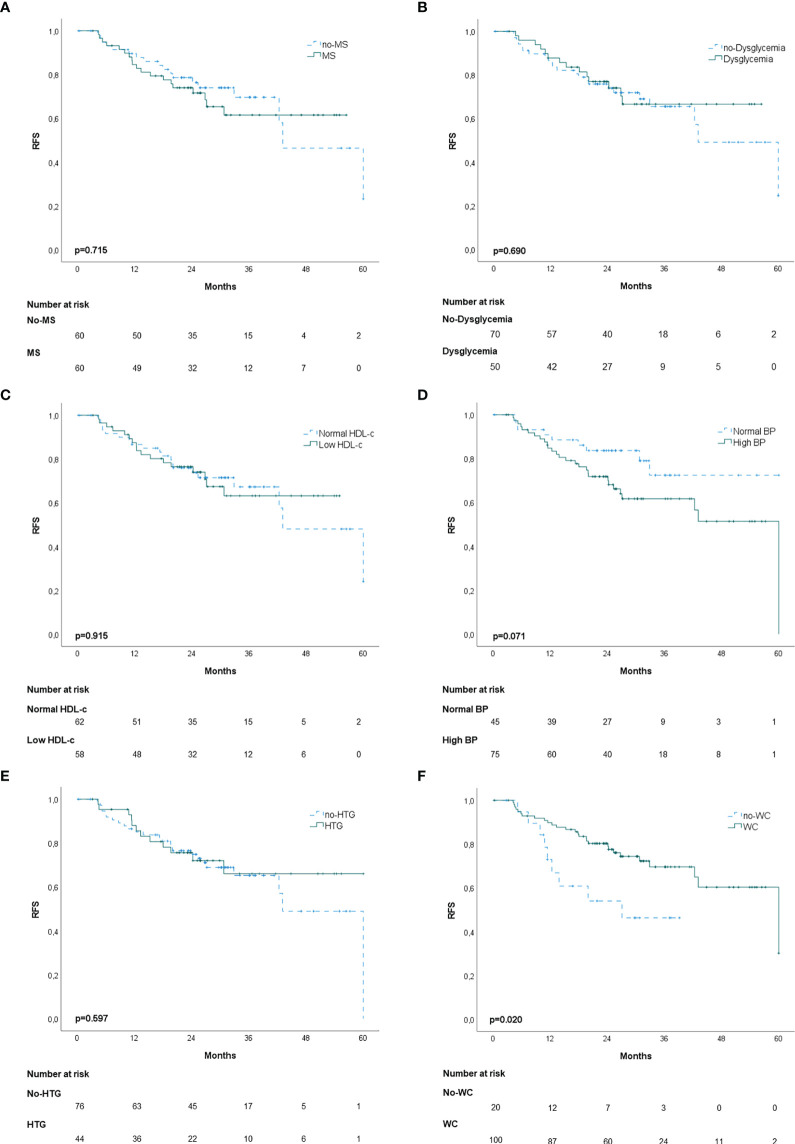
Kaplan-Meier recurrence-free survival curves (RFS): **(A)** patients with and without metabolic syndrome (MS); **(B)** patients with and without dysglycemia (fasting plasma glucose ≥100 mg/dl); **(C)** patients with and without low HDL-c; **(D)** patients with and without high blood pressure (BP); **(E)** patients with and without hypertriglyceridemia; **(F)** patients with and without elevated waist-circumference (WC).

In the propensity score‐matching analysis, 60 patients in the MS group and 60 patients in the no‐MS group were matched and analyzed. There was no statistical significance in the adjusted covariates of the 120 patients after PS matching (**Supplementary Table 2**). Balance between groups could also be observed in the histograms of the propensity scores before and after the matching (**Supplementary Figure 1**). The Kaplan-Meier analysis showed no statistical significance in OS curves for MS or its individual components (**Figure 1**). High BP was associated with worse DFS (p=0.020) (**Figure 2D**) but no differences were observed in RFS (p=0.071) (**Figure 3D**). In contrast, elevated WC was associated with improved DFS (p=0.003) (**Figure 2F**) and RFS (p=0.020) (**Figure 3F**). No other statistically significant differences were found in the Kaplan-Meier survival curves (**Figures 2A–C, E** and **Figures 3A–C, E**). Data on the effects of MS and its components as prognostic factors for OS, RFS and DFS are provided in **Table 4**. Univariate Cox regression analysis revealed that high BP [hazard ratio (HR), 2.11; 95% confidence interval (CI), 1.11 –4.04] and WC [hazard ratio (HR), 0.40; 95% confidence interval (CI), 0.21 –0.75] were independent prognostic factors for DFS (**Table 4**). After multivariate analysis both variables retained significance (**Table 4**).

**Table 4:** Univariate survival analysis of MS and components for RFS, DFS and OS of the matched patients.

Variable	OS		DFS		DFS		RFS	
	Univariate		Univariate		Multivariate		Univariate	
	HR (95% CI)	p value	HR (95% CI)	p value	HR (95% CI)	p value	HR (95% CI)	p value
MS	1.34 (0.67–2.67)	0.412	1.08 (0.62–1.87)	0.793	–	–	1.13 (0.59–2.18)	0.715
Dysglycemia	1.64 (0.83–3.25)	0.153	1.05 (0.61–1.84)	0.853	–	–	0.87 (0.45–1.71)	0.690
Low HDL-c	1.28 (0.64–2.52)	0.500	1.07 (0.62–1.86)	0.813	–	–	0.97 (0.50–1.86)	0.915
High BP	1.36 (0.63–2.94)	0.433	2.11 (1.11–4.04)	**0.023**	2.12 (1.11–4.05)	**0.023**	1.98 (0.93–4.20)	0.076
Hypertriglyceridemia	1.06 (0.52–2.16)	0.869	0.85 (0.45–1.53)	0.594	–	–	0.83 (0.42–1.66)	0.597
WC	0.62 (0.29–1.35)	0.228	0.40 (0.21–0.75)	**0.005**	0.39 (0.21–0.75)	**0.004**	0.41 (0.19–0.89)	**0**.**024**

BP, Blood pressure; DFS, Disease-free survival; HR, Hazard ratio MS, Metabolic Syndrome; OS, Overall survival; RFS, Relapse-free survival; WC, waist circumference. In bold: p-values with statistical significance.

## Discussion

Epidemiological data showed that MS and individual MS components confer a higher risk for adenoma and CC (4, 9–11). In fact, several inflammatory cytokines and adipokines associated with abdominal obesity and insulin resistance were hypothesized to be the pathophysiological link between MS and CC (3, 12). In this study, we aimed to evaluate whether the presence of MS or individual MS components could predict CC tumor characteristics and disease prognosis.

Our results show an association between MS and early T-stage (35.3 vs 14.5%, p=0.002). When assessing the impact of each individual component, only low HDL-c maintained this association (p=0.008). Of notice, all patients in this group were under lipid-lowering drugs (statins, fibrates or other). As elevated total cholesterol and LDL-c levels were demonstrated to have deleterious effects in non-metastatic CRC survival (13), a possible protective role for statins and other cholesterol-lowering agents, such as ezetimibe, has been suggested (14, 15). In fact, the long-term use of statins has been associated with less advanced tumor stage, lower frequency of distant metastasis and better overall survival (16). The mechanism of action of cholesterol-lowering agents in CC is still unclear. Despite the fact that statins and ezetimibe were shown to interfere with several cancer-related processes such as inflammation, cell proliferation, angiogenesis, apoptosis, and metastization, recent studies failed to demonstrate that statins improve CC survival, regardless of KRAS mutation status (17, 18).

Our primary findings also revealed a smaller tumor size in the MS group (4.0 vs 4.5 cm, p=0.006), particularly in the group of patients with high BP (p=0.029). A possible explanation for these results could be related to the recently demonstrated anti-proliferative and apoptotic potential of antihypertensive drugs, such as angiotensin-converting-enzyme inhibitors (ACEi) and angiotensin receptor blockers (ARB) (19–22). In fact, there are several common links between hypertension and tumor development, since angiogenesis and oxidative stress play an important role in both conditions by modulating inflammation, vascular tone, cell growth and differentiation (23). Chen et al. reported that the ACEi captopril and the ARB losartan/irbesartan induced a dose-dependent inhibition of cell proliferation and VEGF secretion in esophageal squamous carcinoma cell lines (20). In addition, Alhusban et al. demonstrated that clinically relevant doses of candesartan inhibited growth of prostate tumor xenografts in mice by decreasing VEGF expression *via* AT1 inhibition. Furthermore, candersartan was demonstrated to reduce vascular lumen size and increase vessel wall thickness, inhibiting tumor vascular permeability and perfusion, impairing neovascularization and thus nutrient supply (22).

In this study, a marginally lower number of LN was retrieved in the MS group (14 vs 16; p=0.052), in accordance with previous reports where patients without MS were more likely to have ≥12 LN retrieved (24). This is likely related to the fact that visceral obesity is an important contributor to increased surgical difficulty by limiting accessibility to LN located deep in the adipose tissue surrounding the major vessels (25). Previous findings support that the extent of lymphadenectomy may influence the pathologic N stage, as the number of positive LN increases in proportion to the number of dissected nodes (26). Therefore, in addition to nodal invasion, several studies also evaluated the prognostic value of LNR as a surrogate endpoint regardless of total nodes in CRC outcomes (26, 27). However, the existing studies regarding the effect of MS in CC outcome focus primarily on the number of dissected LN (24, 28). For that reason, to our knowledge the impact of MS on LNR has not been addressed. Still, we found no association between MS and the extent of nodal involvement according to metastatic LN (p=0.277) or LNR (p=0.332).

No significant relationship was identified between MS and other tumor pathological features, such as tumor primary location, pathological staging and local or distant invasion. These findings are in accordance with several previous studies conducted in this field (13, 28, 29).

In this study, we found no association between MS or any individual MS component and CC OS. An inconsistent association between MS and its individual components on CC mortality and disease recurrence has been reported in previous epidemiologic studies. A large retrospective cohort of 36.079 patients with CC found no association of MS with OS or RFS. However, the authors attributed this result to the impact of individual components since diabetes and hypertension decreased survival, whereas dyslipidemia revealed a protective effect improving OS and RFS (24). In another study, MS, diabetes and hypertension had no prognostic impact in OS in non-metastatic CRC patients. On the contrary, patients without MS had improved DFS (p=0.014) and cumulative 3-, and 5-year DFS (p=0.039, p=0.044, respectively) (13). These findings seem to suggest that, regardless of more favorable tumor characteristics, co-morbidities associated with MS, such as dysglycemia, can have a deleterious effect on survival. In our study, patients with higher WC showed improved DFS and RFS. Since a large percentage of patients with high-WC were under treatment with lipid lowering drugs, this result suggests that DFS improvement could be related to the protective effect of these agents as discussed above. Nevertheless, despite lipid lowering agents being suggested to have beneficial effects by limiting tumor infiltration these may not have the necessary robustness to influence CC prognosis, as OS was not affected. Previous studies focusing on the potential role of obesity in CRC survival has yielded heterogeneous results. Additionally, recent results revealed that a normal weight could be protective for cardiovascular death, but not CRC recurrence (30). Despite its possible association with smaller tumor size at diagnosis, high BP demonstrated to negatively affect DFS, revealing as an independent prognostic factor after multivariate analysis [hazard ratio (HR), 2.12; 95% confidence interval (CI), 1.11 –4.05]. Hypertension is a known side effect of all angiogenesis inhibitors used in the treatment of CC (31). Therefore, for individuals with high BP at baseline, it may act as a barrier to the initiation of such treatment, dose restrictions or premature discontinuation, and thus affecting DFS.

The conflicting results on the effect of MS in CC outcomes reported by clinical studies may be explained by the different definitions of MS in different populations. Since insulin resistance was thought to be one of the major pathophysiological mechanisms of MS, this remained a precondition for the World Health Organization (WHO) definition of MS (32). Likewise, since central obesity is also considered one of the key elements for metabolic disorders the International Diabetes Federation (IDF) considers abdominal obesity a prerequisite for MS diagnosis (33). Thus, the use of WHO and IDF criteria may lead us to conclude that a non-diabetic or non-overweight patient is healthy, although it is possible that the individual is metabolically unhealthy. The National Cholesterol Education Program (NCEP) Adult Treatment Panel III (ATP III) and the Harmonized Criteria of the Joint Interim Societies require that patients meet at least 3 of the 5 MS criteria and unlike WHO or IDF definitions, do not require any specific criteria (5, 34). The Harmonized Criteria and IDF use population-specific cut-off points for WC measurement (5, 33). Additionally, several studies performed in the Asian population use the criteria defined by the Chinese Diabetes Society (35). These criteria differ from those described above in several aspects, including the use of body mass index as a measure of obesity, the merging of lipid deviations into one criterion and the use of different cutoff values for blood pressure, glucose and HDL-c (35). Conflicting results may also arise from the fact that most studies fail to control for cancer type (i.e., colon vs rectum) and treatment, which can particularly affect survival analysis.

Our results must also be interpreted in the light of study limitations. First, this was a retrospective single-center study, which constrains sample size and may cause some selection bias. Therefore, some predictive associations may have been missed due to sample size that hampers the robustness of our conclusions and limits generalization of the conclusions. Second, the impact of different treatment plans for MS individual components on patient outcome could not be determined. This can be an important source of bias, since most patients under treatment for dyslipidemia were treated with statins, which target mostly LDL-c and HDL-c in a smaller extension. Third, due to the retrospective nature of the study, additional potential confounding factors such as family history of cancer, cigarette smoking, alcohol consumption, dietary habits and physical activity were not evaluated. Data on these factors would be of great interest since these are known to play a dual role in the pathogenesis of metabolic syndrome and cancer, which may affect survival. In addition, since patients are referred to our institution from primary care centers for elective treatment, data on whether CRC diagnosis was due to screening or symptoms driven, could not be collected. This relevant information if available could expand the possibility of hypothesis testing. A further limitation of our study may be the lack of gender stratification since some data suggest a higher risk and worse outcome in men (4, 10, 28).

Despite these limitations, our study provides a better understanding of the link between MS and CC phenotype and outcome. Although several studies point to a higher risk of CC in metabolically dysfunctional patients, this deleterious effect on tumor aggressiveness could have been counterbalanced by the protective effect of statins and ACEi/ARB. This study provides relevant information for routine clinical practice, since this applies to a considerable proportion of patients with CC that also present MS and are under blood pressure, lipid and glucose lowering drugs. Further investigations should be designed using prospective clinical data with extensive data on medication, weight variation and clinical parameters such as blood pressure, glucose and lipid serum levels, to extend our findings and also address gender-related influences.

In conclusion, despite the recognized relationship between several MS components and cancer risk, our data shows that patients with this metabolic dysfunction seem to have more favorable tumor characteristics, such as primary tumor diameter and disease extension. Therefore, our findings do not support the hypothesis that MS has a negative impact on CC prognosis. However, given the fact that most patients with MS were under treatment with BP and lipid lowering drugs, the potential influence of the protective role of these pharmacological agents cannot be excluded.

## Data Availability Statement

Research data are not publicly available due to privacy and ethical restrictions. However, anonymized data that are required to reproduce results can be made available from the corresponding author upon reasonable request, and upon approval from the Centro Hospitalar Universitário do Porto according to mandatory national law.

## Ethics Statement

The studies involving human participants were reviewed and approved by Comissão de Ética do Centro Hospitalar Universitário do Porto. Written informed consent for participation was not required for this study in accordance with the national legislation and the institutional requirements.

## Author Contributions

Conceptualization, AS, MM, and GF. Methodology, AS, MM, and GF. Formal analysis, AS and SP. Investigation, AS. Data curation, AS. Writing—original draft preparation, AS. Writing—review and editing, AS, SP, MM, AA, and GF. Supervision, MM and GF. Project administration, MM and GF. All authors contributed to the article and approved the submitted version.

## Conflict of Interest

The authors declare that the research was conducted in the absence of any commercial or financial relationships that could be construed as a potential conflict of interest.
