# Supplementary material for: Effect of Metabolic Syndrome and Individual Components on Colon Cancer Characteristics and Prognosis
Source: Front Oncol. 2021 Mar 4;11:631257. doi: 10.3389/fonc.2021.631257 (PMC7970759; doi:10.3389/fonc.2021.631257)
Supplement: Supplementary file 1 [file DataSheet_1.pdf]

Supplementary Material

Supplementary Table 1 - AJCC classification according to metabolic syndrome status

	All patients (n=168)	No MS (n=83)	MS (n=85)	p value
AJCC Classification, n (%)				0.285
I	36 (21.4)	11 (13.3)	25 (29.4)	
IIa	41 (24.4)	25 (30.1)	16 (18.8)	
IIb	4 (2.4)	2 (2.4)	2 (2.4)	
IIc	3 (1.8)	1 (1.2)	2 (2.4)	
IIIa	5 (3.0)	2 (2.4)	3 (3.5)	
IIIb	39 (23.2)	21 (25.3)	18 (21.2)	
IIIc	8 (4.8)	5 (6.0)	3 (3.5)	
IVa	17 (10.1)	10 (12.0)	7 (8.2)	
IVb	15 (8.9)	6 (7.2)	9 (10.6)	

AJCC – American Joint Committee on Cancer; MS – Metabolic syndrome

Supplementary Table 2 - Characteristics of propensity-score matched patients before and after matching

	Before matching			After matching		
	**No MS (n=83)**	**MS (n=85)**	**p value**	**No MS (n=60)**	**MS (n=60)**	**p value**
Age (years), mean (SD)	67 (14)	74 (9)	<0.001	72 (12)	72 (9)	1.000
Tumor size (cm), median (IQR)	4.5 (3.5-5.5)	4.0 (3.0-5.0)	0.006	4.1 (3.5-5.5)	4.0 (3.0-5.5)	0.348
T stage, n (%)						
1/2	12 (14.5)	30 (35.3)	0.002	11 (18.3)	13 (21.7)	0.648
3/4	71 (85.5)	55 (64.7)		49 (81.7)	47 (78.3)	
N stage, n (%)						
0	48 (57.8)	54 (63.5)	0.185	34 (56.7)	36 (60.0)	0.870
1	19 (22.9)	23 (27.1)		16 (26.7)	16 (26.7)	
2	16 (19.3)	8 (9.4)		10 (16.7)	8 (13.3)	
M stage, n (%)						
0	66 (79.5)	69 (81.2)	0.787	46 (76.7)	49 (81.7)	0.500
1	17 (20.5)	16 (18.8)		14 (23.3)	11 (18.3)	

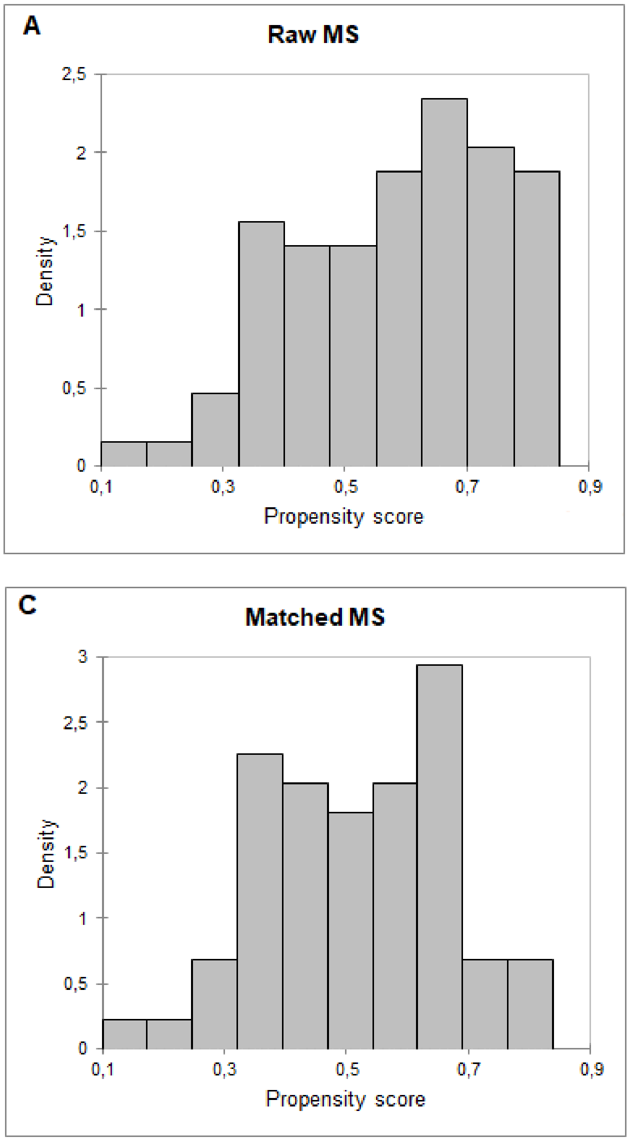

Supplementary Figure 1 - Histograms of propensity scores before and after matching
